# The association between hormonal contraceptive use and smoking, negative affect, and cessation attempts in college females

**DOI:** 10.1016/j.dadr.2022.100063

**Published:** 2022-05-11

**Authors:** Shelby A. Stewart, MacKenzie R. Peltier, Melanie R. Roys, Amy L. Copeland

**Affiliations:** a Department of Psychology, Louisiana State University Baton Rouge, LA 70803, USA; b Department of Psychiatry, Yale School of Medicine, USA; c Psychology Service, VA Connecticut Healthcare System, USA; d Pennington Biomedical Research Center, USA

**Keywords:** Hormonal contraceptives, Progestin, Female smokers

## Abstract

Exogenous and endogenous female hormones influence nicotine use and cessation, potentially through mechanisms such as anxiety and negative affect. In the present study, college females using all types of hormonal contraceptives (HC) were compared to those not using HC to determine the potential influence on current smoking, negative affect, and current and past cessation attempts. Differences between progestin-only and combination HC were also examined. Of the 1,431 participants, 53.2% (*n* = 761) reported current HC use, and 12.3% (*n* = 176) of participants endorsed current smoking. Women currently using HC were significantly more likely to smoke (13.5%; *n* = 103) compared to women not using HC (10.9%; *n* = 73), *p* = .04. There was a significant main effect of HC use being associated with lower anxiety levels (*p* = .005), as well as a significant HC use by smoking status interaction, such that women who smoke using HC reported the lowest levels of anxiety among participants (*p* = .01). Participants using HC were more likely to be making a current attempt to quit smoking than those not using HC (*p* = .04) and were more likely to have made past quit attempts (*p* = .04). No significant differences were observed across women using progestin-only, combined estrogen and progestin, and women not using HC. These findings provide evidence that exogenous hormones may be an advantageous treatment target and that they warrant additional study.

## Highlights

• Women currently using hormonal contraceptives were more likely to smoke.
• Smokers using hormonal contraception reported the lowest anxiety levels.
• Hormonal contraception was associated with current and past cessation attempts.
• Exogenous hormones may be an advantageous cessation treatment target.

Prevalence rate estimates for cigarette smoking among women are lower (12.0%) than among men (15.6%) in the United States (Creamer et al., 2019). However, despite smoking at lower rates, women are more vulnerable than men to smoking-related illnesses, such as heart disease and lung cancer (Allen et al., 2014; Huxley and Woodward, 2011; Kiyohara and Ohno, 2010). Given this added vulnerability, it is critical for women to be able to quit smoking successfully, as cessation has been associated with a myriad of health benefits for both men and women who smoke (Allen et al., 2014; Frank, 1993; Goldman et al., 2009). Women, however, have more difficulty quitting smoking than men and have consistently exhibited lower abstinence rates following smoking cessation treatment (Bjornson et al., 1995; Smith et al., 2016; Wetter et al., 1999b). Studies indicate that nicotine withdrawal and the cessation process itself may be particularly challenging for women who smoke, as they tend to smoke to alleviate negative affect (Faulkner et al., 2018; Leventhal et al., 2007; Pang and Leventhal, 2013). A further hindrance to cessation efforts among women, is evidence that they may be less responsive to nicotine replacement therapy as a cessation aid (Perkins and Scott, 2008; Weinberger et al., 2015; Wetter et al., 1999a). Existing sex differences in smoking cessation may be attributed to a variety of psychosocial and biological variables specific to women (Sieminska and Jassem, 2014).

These sex differences may be due in part to the influence of ovarian hormones, including estradiol and progesterone, which undergo significant fluctuations throughout the natural menstrual cycle in premenopausal women. A normal ovarian cycle has two major stages: (1) the follicular phase, comprising the beginning of the cycle to ovulation; and (2) the luteal phase, which occurs between ovulation and the next menstruation (Patricio and Sergio, 2019). These phases correspond with changing levels of estradiol and progesterone. Estradiol levels increase during the middle follicular phase, decrease rapidly after ovulation, then further increase in the mid-luteal phase. During this same time in the luteal phase, increasing quantities of progesterone are produced. In the absence of pregnancy, at the end of the luteal phase, progesterone and estradiol levels decrease and a new menstrual cycle begins (Knudston and McLaughlin, 2019). These hormonal fluctuations can influence nicotine self-administration (Allen et al., 2008; Pomerleau et al., 1992), nicotine craving and withdrawal (Carpenter et al., 2006), and post-cessation relapse (e.g., Allen et al., 2018a; Allen et al., 2009a).

Studies have attempted to determine which phase of the cycle is most conducive to cessation attempts among women who smoke and thereby identify a role for ovarian hormones. Some studies found a higher relapse risk in the follicular phase, potentially due to estrogen's influence on nicotine and mood (Allen et al., 2009b; Allen et al., 2008). Other studies have found higher abstinence rates among women who quit in the follicular phase who are also using nicotine replacement therapy (NRT; Carpenter et al., 2008; Franklin et al., 2008). Discrepant findings in this literature have often been attributed to methodological differences in defining the dichotomous phases of the menstrual cycle (Franklin and Allen, 2009; Allen et al., 2016), as well as interactions with pharmacotherapeutic cessation aids, such as NRT (Allen et al., 2009b), and the metabolic effects of ovarian hormones on nicotine (Benowitz et al., 2006). Research has speculated, however, that female sex hormone affect smoking by means of their effects on mood.

Endogenous female sex hormones have been shown to influence mood. For example, there is evidence that endogenous estrogen has antidepressant and anxiolytic effects (e.g., Ma et al., 2020; Hughes et al., 2008). Anxiety appears to fluctuate with the phase of the menstrual cycle, and anxiety symptoms have been associated with low endogenous estradiol levels (Borrow and Handa, 2017). The evidence regarding progesterone's effect on mood is more varied, with some women experiencing negative mood symptoms during the luteal phase of the menstrual cycle when progesterone levels are elevated, or while using hormonal contraceptives (HC) that contain progestogens (Andréen et al., 2009; Weinberger et al., 2015). However, other evidence indicates that progesterone reduces negative affect, which is especially important as women who smoke are motivated to smoke for relief of negative affect and stress management (Perkins 1996; Perkins, 2001; Saladin et al., 2012).

Exogenous female sex hormones, such as those contained in widely used hormonal contraceptives (HC), provide a method to study hormonal influence on smoking behavior, negative affect, and cessation among women who smoke. It is also important to understand the influence of HC on smoking, as it is estimated that 27% of premenopausal women take some form of HC (McClave et al., 2010). Oral contraceptives, for example, are typically combined preparations of estrogen plus progestin. They may be monophasic, providing low constant levels of both hormones, or bi- or tri-phasic, providing variable levels of estrogen and progestin over the course of a menstrual cycle (Hall and Trussell, 2012). Progestin refers to types of synthetic progestogens, or progesterone, such as MPA, norethindrone, and levonorgestrel which are commonly found in hormonal birth control (Stanczyk et al., 2013). These synthetic progestins are not bioidentical to progesterone and react differently with progesterone receptors in the body; they result in lower endogenous progesterone and estradiol levels by preventing ovulation (Montoya and Bos, 2017; Wiegratz and Kuhl, 2006).

Research has accumulated on the effects of HC and smoking cessation among women. Some studies have identified greater success with short-term abstinence among women who use HC versus those who don't, as well as smoking-related withdrawal symptomatology that is distinct from that in women who don't use HC (Allen et al., 2018b; Hinderaker et al., 2015). Allen et al. (2019) found that women who used combination HC experienced significantly worse nicotine withdrawal effects, cravings, and negative affect when making a cessation attempt. Other studies have similarly found HC to be associated with adverse mood effects (Skovlund et al., 2016, 2018). Still other studies have not found clinically significant effects of HC on mood (Graham et al., 1995; O'Connell et al., 2007). It is therefore possible that the observed negative effects of HC in studies were due to uncontrolled factors such as psychosocial stress, family history of depression, comorbid medical or substance abuse problems, or differential effects of hormonal preparations on the central nervous system (Robakis et al., 2019). Arguably, HC's effects of stabilizing the typical hormonal fluctuations observed across the menstrual cycle and consequent reduced fluctuations in affect and stress, indicate that HC use could potentially yield advantageous cessation outcomes among women who smoke. This argument is especially compelling, given the established association between negative affect and smoking among women.

Few existing studies have investigated the relationship among exogenous female sex hormones, smoking behavior, negative affect, and cessation attempts among premenopausal women. Better understanding of how female sex hormones influence smoking-related variables in premenopausal women who smoke could inform cessation intervention efforts for this population. We conducted the current study with the overall goal of determining the influence of HC on smoking behavior, including cessation, and negative affect, as it has been identified as a motive for smoking and relapse, especially among women who smoke. The specific aims of the present study were to (1) estimate the prevalence of HC use and type among premenopausal college women; (2) identify the nature of the influence HC use has on smoking-related behavior, including smoking rate, negative affect (including anxiety), and cessation attempts; and (3) explore whether this influence differs by HC type (progestin-only versus combined estrogen and progestin). Specifically, we hypothesized that women who smoke would report experiencing higher levels of negative affect as compared to those who don't smoke, but that women who smoke and use HC would report lower negative affect than women who smoke and don't use HC. Second, we hypothesized that women who smoked and were using HC would be more likely to be currently attempting to quit smoking and have more past cessation attempts. Lastly, we hypothesized that if hypotheses 1 and 2 were supported, further exploratory analysis would show significant differences in how the various types of hormonal contraception (i.e., progestin-only HCs and combination estrogen and progestin HCs) would influence negative affect and current and past cessation attempts.

## 1 Methods

### 1.1 Participants

Participants were recruited through the online registration system for the psychology department experiment participant pool at a large southern university. The study inclusion criteria listed on the registration site required that participants be: (a) female; (b) currently enrolled as an undergraduate student at the university at which the study was being conducted; and (c) between 18 and 24 years of age. Despite the listed inclusion criteria, 24 participants reported being over 24 years of age and were therefore excluded from analyses, yielding the sample size of 1431. All 1455 participants were granted course extra credit for participating.

### 1.2 Procedure

This research was reviewed and approved by the university's Institutional Review Board prior to data collection. Participants were recruited from the psychology department's research participant pool and granted course extra credit for participation. Participants provided informed consent and completed a series of measures via a secure online survey engine. Participants received extra credit points after exiting the survey regardless of level of completion of survey. Measures included a smoking status and demographic questionnaire, anxiety and general negative affect measures, and questions regarding their hormonal contraception use. All participants (*N* = 1431) completed demographic and smoking status information, and at least one measure of mood and were therefore included in the statistical analyses.

### 1.3 Measures

**Fig. 1:**
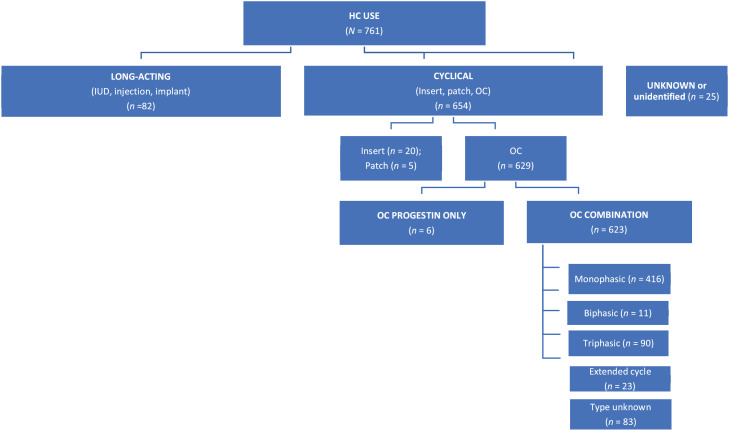
Types of HC reported by participants.
*State-Trait Anxiety Inventory* (STAI; Spielberger, 1983). The State-Trait Anxiety Inventory (STAI) is a commonly used measure of trait and state anxiety. The STAI (Spielberger, 1983) is a self-report instrument designed to assess levels of state anxiety and trait anxiety with 40 items scored by a Likert-scale. State anxiety can be defined as a transient momentary emotional status that results from situational stress. Trait anxiety represents a predisposition to react with anxiety in stressful situations. The STAI includes two subscale scores, state anxiety and trait anxiety. Scores are calculated by separately summing scores for the state items and the trait items. Total scores range from 0–63. The following guidelines are recommended for the interpretation of scores: 0–9, normal or no anxiety; 10–18, mild to moderate anxiety; 19–29, moderate to severe anxiety; and 30–63, severe anxiety. Internal consistency alpha coefficients have ranged from 0.86 to 0.95 (Spielberger, 1983). Considerable evidence attests to the construct and concurrent validity of the scale (Spielberger, 1989).
*Positive and Negative Affect State* (PANAS; Watson et al., 1988). The PANAS is a 20-item self-report measure that assesses an individual's positive affect (PA) and negative affect (NA) at a given point in time. Twenty different emotions are listed, and individuals rate items on a 5-point Likert scale. Positive and negative affect items are summed separately, yielding 2 scores that range from 10 to 50. Higher scores indicate greater levels of positive and negative affect, respectively. The PANAS has shown good internal consistency (*r* = 0.86 - 0.90 for PA and *r* = 0.84 - 0.87 for NA) and moderate concurrent validity (*r* = 0.51 - 0.74).

*Smoking Status Questionnaire.* This form included demographic questions, such as age and ethnicity, and smoking-related variables, such as current and past smoking patterns, and current and past cessation attempts. This form also included the Fagerström Test for Cigarette Dependence (FTCD; Fagerström, 2012) to assess nicotine dependence level in persons who smoke daily. The items are summed to yield a total score of 0–10. The higher the total Fagerström score, the more intense is the individual's physical dependence on nicotine, with scores of 0 to 2 = very low dependence, 3 to 4 = low dependence, 5 = medium dependence, 6 to 7 = high dependence, and 8 to 10 = very high dependence. The FTCD has acceptable levels of internal consistency and is closely related to biochemical indices of heaviness of smoking (Fagerström, 2012).
*Hormonal Contraception Information*. Participants were asked the following questions: Are you currently using hormonal contraception (HC) (yes/no)? If yes, what is the name of the HC you currently take? The latter question was intentionally left open-ended, so that the investigators could reference the HC name, type, and exogenous hormone activity (e.g., progestin only, combined estrogen and progestin) to be coded as described in Fig. 1.

### 1.4 Data analytic strategy

Preliminary analyses of descriptive characteristics were conducted which allowed for identification of covariates. Chi square analyses were conducted with dichotomous or categorical dependent variables, and analyses of variance (ANOVAs) and multivariate analyses of variance (MANOVAs) were conducted with continuous dependent variables with HC use (yes/no) and smoking status (yes/no) as factors.

To test the first hypothesis (women who smoke would report higher negative affect than women who don't smoke, and women who smoke and use HC would report lower negative affect than women who smoke but don't use HC), we conducted a two-way factorial multivariate analysis of covariance (MANCOVA) with HC use and smoking status as the factors, STAI state and trait anxiety scores as dependent variables, and age which was identified as a covariate. We conducted another two-way factorial MANCOVA with HC use and smoking status as the factors, the PANAS Positive and Negative affect scales as dependent variables, and age as a covariate. Next, to test the second hypothesis (women who smoked and were using HC would be more likely to be attempting to quit smoking and to have more past cessation attempts than smokers not using HC), we conducted a chi-square analysis with smokers only and with HC use and current cessation attempt (yes/no) as the factors.

Finally, to test the third hypothesis (progestin-only HCs would differ from combined estrogen and progestin HCs in their influence on negative affect and current and past cessation attempts, we conducted a one-way ANCOVA with HC type (none, progestin-only, combined estrogen and progestin) as the factor, age as a covariate, and STAI state anxiety scores as dependent variables. We then followed up with least standard difference (LSD) post-hoc tests to identify significant between-group differences. A chi-square analysis with HC type and cessation attempt (yes/no) as the factors was conducted, and paired comparison post-hoc tests were used to identify between-group differences.

Armstrong (2014) argues that Bonferroni corrections should not be used routinely, mainly due to the consequent risk in Type 2 Error that can occur with setting a more stringent alpha level. He suggests the Bonferroni correction be considered if: (1) a single test of the 'universal null hypothesis' (H_o_) that all tests are not significant is required; (2) it is imperative to avoid a type I error; and (3) a large number of tests are carried out without pre-planned hypotheses. We therefore applied a Bonferroni correction to the post-hoc tests we conducted as follow up to analyses related to our three primary hypotheses. In interpreting those results, alpha was adjusted to *p* < .017.

## 2 Results

### 2.1 Participant characteristics

There were 1431 participants, who were predominantly Caucasian (78.0%) and 13.8% African American, with a mean age of 20.0 (*SD* = 2.0). Among the 12.3% (*n* = 176) who endorsed current smoking, 28.4% (*n* = 50) endorsed smoking daily and reported a mean smoking rate of 6.6 (*SD* = 5.1) cigarettes per day (CPD), for a mean of 3.6 (*SD* = 3.4) years, and mean FTCD score of 1.7 (*SD* = 1.9). Those women who endorsed nondaily smoking (*n* = 126) reported smoking an average of 2.1 (*SD* = 3.1) cigarettes per week, for an average of 2.6 (*SD* = 2.0) years. See Table 1 for participant characteristics by HC use status and smoking status.

**Table 1:** Participant characteristics by HC use and smoking status.

	HC Users (*n* = 761)		Non-HC Users (*n* = 670)		
	Nonsmoker (*n* = 658)	Smoker (*n* = 103)	Nonsmoker (*n* = 597)	Smoker (*n* = 73)	*p*
Age	19.95 (*1.59*)_a_	20.03 (*1.72*)_b_	19.91 (*2.32*)_c_	20.75 (*2.45*)_abc_	.007
% Caucasian	91.5%	91.3%	89.4%	90.8%	.363
STAI State Anxiety	44.85 (*8.16*)_a_	42.80 (*8.92*)_abc_	45.05 (*8.10*)_b_	46.58 (*7.77*)_c_	.029
STAI Trait Anxiety	47.58 (*7.13*)	47.10 (*7.50*)	47.32 (*6.94*)	48.94 (*5.82*)	.311
PANAS Pos. Affect	26.42 (*9.17*)	25.51 (*9.54*)	26.03 (*9.05*)	24.81 (*8.24*)	.477
PANAS Neg. Affect	16.40 (*6.31*)	16.40 (*6.75*)	15.98 (*6.79*)_a_	17.84 (*7.93*)_a_	.07
Weekly smoking rate	–	13.17 (*25.81*)	–	11.06 (*22.43*)	.643
# Years smoking	–	2.86 (*2.03*)	–	3.35 (*2.85*)	.189
# Quit attempts*	–	3.14 (*10.84*)	–	1.80 (*4.12*)	.349
Daily smoking rate	–	5.82 (*4.41*)	–	7.41 (*5.62*)	.112
FTCD (daily smokers only)	–	1.39 (*1.45*)	–	2.08 (*2.25*)	.157

Note: HC = hormonal contraception; STAI = Spielberger State/Trait Anxiety Inventory; PANAS = Positive and Negative Affect Schedule; Pos. = Positive; Neg. = Negative; * indicates # of quit attempts in lifetime; FTCD = Fagerström Test for Cigarette Dependence. Subscript letters indicate significant between group differences.

Seven hundred sixty-one participants (53.2%) reported current HC use and reported the name of the HC (e.g., Nuva ring, Yaz), which was then coded by the experimenters into the categories as outlined by Allen et al. (2018b) in their classification system for HC. Eighty-two participants reported using long-acting HC such as an intrauterine device (IUD), injection, or implant. Among these participants, 8 reported use of an IUD, 44 reported use of an injection, 23 reported use of an implant, and the remaining 7 participants did not know their type of long-acting HC. It should be noted that no participants reported using a copper IUD (i.e., nonhormonal). Six hundred fifty-four participants reported using a cyclical HC method, such as oral contraception (OC), insert, or patch. The remaining 25 participants endorsed current HC use but could not recall the name of their prescription and were therefore excluded from subsequent analyses since HC type was unknown. Among the participants reporting use of cyclical HC, 20 reported use of an insert, 5 reported use of a patch, and 629 reported use of OC. Among the participants reporting OC, 6 reported use of progestin-only OC, and 623 reported using OC that was a combination of estrogen and progestin. These combined OCs were further divided into the categories of monophasic (*n* = 416), biphasic (*n* = 11), triphasic (*n* = 90), extended cycle (*n* = 23), and unknown type (*n* = 83). See Fig. 1. Participants using the long-acting, progestin-only HCs (*n* = 82) formed a progestin-only group in subsequent analyses. Participants using progestin-only OCs (*n* = 6) were excluded from subsequent analyses, given differences in symptoms associated with these HC types and the potential impact this may have on smoking-related outcomes.

### 2.2 Smoking status among HC users

Women currently using HC (including long-acting) were more likely to be smoking (13.5%; *n* = 103) as compared to women not currently using HC (10.9%; *n* = 73), *X*^2^(1) = 2.30, *p* = .04, *phi* = 0.08. Next, two-way factorial analyses of variance (ANOVAs) indicated a main effect for HC use *F*(1, 1424) = 4.48, *p* = .034, *η_p_^2^* = 0.003, such that HC users were significantly younger (*M* = 20.0; *SD* = 1.6) than non-HC users (*M* = 20.0; *SD* = 2.4). There was a significant main effect for smoking *F*(1, 1424 = 8.14, *p* = .004, *η_p_^2^* = 0.006, such that smokers were significantly older (*M* = 20.3; *SD* = 2.08) than nonsmokers (*M* = 19.9; *SD* = 2.0). There was also a significant smoking status by HC use interaction, *F*(1, 1424) = 5.62, *p* = .018, *η_p_^2^* = 0.004, whereby older women who smoked were significantly less likely to use HC as compared to women who didn't smoke of any age. Age was therefore used as a covariate in subsequent analyses. There were no significant differences in weekly or daily smoking rate between women who smoked and used HC and those not using HC. See Table 1 for descriptive statistics by smoking status and HC use group.

### 2.3 Smoking, HC use, negative affect

The overall MANCOVA was significant for HC use [Wilks’ Lambda = 0.99, *F*(2, 1427) = 3.95, *p* = .02, *η_p_^2^* = 0.007] and for a HC use by smoking status interaction [Wilks’ Lambda = 0.99, *F*(2, 1427) = 3.54, *p* = .029, *η_p_^2^* = 0.006]. The main effect for HC use on state anxiety was significant*, F*(1, 1132) = 7.90, *p* = .005, *η_p_^2^* = 0.007, with women who used HC having lower state anxiety scores (*M* = 44.6; *SD* = 8.3) as compared to those who were not using HC (*M* = 45.2; *SD* = 8.1). The HC use by smoking status interaction for state anxiety was also significant, *F*(1, 1132) = 6.70, *p* = .010, *η*_p_^2^ = 0.007, such that women who smoked and used HC had the lowest state anxiety scores (*M* = 42.8; *SD* = 8.9). The HC use by smoking status interaction for trait anxiety approached significance, *F*(1, 1132) = 3.09, *p* = .08, *η*_p_^2^ = 0.003, such that women who smoked and used HC had the lowest trait anxiety scores (*M* = 47.1; *SD* = 7.5).

For the PANAS scores, the overall MANCOVA approached significance for smoking status and negative affect [Wilks’ Lambda = 1.0, *F*(2, 1238) = 2.6, *p* = .07, *η*_p_^2^ = 0.004, indicating a trend for women who smoke to have higher negative affect (*M* = 17.0; *SD* = 7.3) than those who didn't smoke (*M* = 16.2; *SD* = 6.6); *F*(1, 1239) = 3.1, *p* = .08, *η*_p_^2^ = 0.002, and for a HC use by smoking status interaction, *F*(1, 1239) = 3.0, *p* = .08, *η*_p_^2^ = 0.002, whereby women who smoked and used HC had lower negative affect scores (*M* = 16.4; *SD* = 6.8) as compared to women who smoked but were not using HC (*M* = 17.84; *SD* = 7.9).

### 2.4 Smoking, hc use, and cessation attempts

Results showed that more participants who were using HC (57.9%) than those not using HC (42.1%) were making a current attempt to quit smoking, *X*^2^(1) = 3.46, *p* = .04, *phi* = 0.080. Participants using HC (62.6%) were also more likely to have made past quit attempts than those not using HC (37.4%), *X*^2^(1) = 3.32, *p* = .04, *phi* = *0*.073. No significant differences were found between women who used HC (*M* = 3.14; *SD* = 10.84) and those who did not (*M* = 1.8; *SD* = 4.1) on number of past quit attempts, *F*(1, 152) = 2.03, *p* = .156, *η*_p_^2^ = 0.013*.*

### 2.5 Progestin-only HC vs. combination hc comparisons among smokers

When participants were separated into groups according to HC type, there were 84 women who smoked in the non-HC group, 11 women who smoked in the progestin-only group, and 80 women who smoked in the combination HC group. There were no significant differences across HC groups on state anxiety scores, *F*(1, 1132) = 1.21, *p* = .30, *η*_p_^2^ = 0.002. State anxiety scores were *M* = 45.8 (*SD* = 8.1) among women using progestin-only HC, *M* = 44.5 (*SD* = 8.2) among women using combination HC, and *M* = 45.2 (*SD* = 8.3) among women not using HC. There were no significant differences across HC groups in current cessation attempt, *X*^2^(2) = 1.45, *p* = .485, *phi* = 0.10, with 16.7% women using progestin-only HC, 26.0% women using combined HC, and 17.9% women not using HC endorsing a current cessation attempt. There were no significant differences across HC groups in past cessation attempt (yes/no), *X*^2^(2) = 3.98, *p* = .136, *phi* = 0.16. Seventy-eight percent of women using progestin-only HC reported having made a past attempt, 42.7% of women using combined HC, and 46.8% of women not using HC had made a past cessation attempt.

## 3 Discussion

In the present sample of premenopausal women, 53.2% reported current HC use, with 44% of the sample reporting oral contraception as the type of HC. This is higher than the estimated 25.3% prevalence of oral contraceptive use among premenopausal women in the United States as well as the estimated 38% of college women who report oral contraceptive use (Coetzee and Ngunyulu, 2015; Kavanaugh and Jerman, 2018). Women who smoke were more likely to be using HC. Among the women who smoked in the current study, HC use was reported at 58.5%, and oral contraceptive use was reported at 50%. This latter figure is almost twice the estimated 27% prevalence of oral contraceptive use alone among premenopausal women who smoke reported elsewhere (e.g., McClave et al., 2010). These differences likely reflect differences between college premenopausal women populations who smoke as compared to premenopausal women who smoke in the general population. To our knowledge, these are the first prevalence rates reported for HC use in college women who smoke. Further research will determine whether these estimates generalize to women who smoke at other universities in various other geographical locations.

As hypothesized, the study found that women who used HC had lower state anxiety scores as compared to women not using HC. In addition, women who smoked and used HC had the lowest state anxiety scores overall. There was a trend in which HC use by smoking status interaction for trait anxiety approached significance in those women who smoked and used HC had the lowest trait anxiety scores. Further, there was a trend toward women who smoked reporting higher negative affect than those who didn't smoke, as well as an interaction by HC use in which women who smoked and used HC reported lower negative affect than women not using HC.

Our present findings indicate that significantly more participants who were using HC (57.9%) than those not using HC (42.1%) were making a current attempt to quit smoking. This is not surprising, as those smoking and using HC had the lowest state anxiety scores and lower levels of negative affect scores. These women may have felt less dependent upon smoking to help regulate stress and negative affect, which has been shown to be especially important in treatment outcomes for women who smoke (McKee et al., 2003). Previous research has identified that women who use combined HC experience significantly worse withdrawal effects, cravings, and negative affect when making a cessation attempt (Allen et al., 2019). Taken together, this illustrates that while women on HC who are smoking may be more motivated to make a quit attempt, these women may also experience more adverse effects during that attempt. Thus, additional research is warranted to target interventions for increased negative affect and stress among women attempting to make a cessation attempt. It should also be noted that women using HC may be more likely to attempt to quit smoking as per recommendation by a healthcare provider, as it's reasonable to assume that the women using HC are engaged with the healthcare system, given they are being prescribed HC. Indeed, healthcare provider recommendations have been shown to lead to smoking quit attempts (Wu et al., 2017).

Finally, the present analyses exhibited no significant differences among varying types of hormonal contraceptives regarding their effect on negative affect and current and past cessation attempts. Women using progestin-only HC were the most likely to have made a past quit attempt (78%), followed by those not using HC (46.8%), followed by those using combined HC (42.7%), but these differences were not statistically significant. There were also no differences across HC group for current cessation attempt, with 16.7% women using progestin-only HC, 26.0% women using combined HC, and 17.9% women not using HC endorsing a current cessation attempt.

It was surprising that there were few significant differences between women using progestin-only HCs, combined HCs, and non-HCs; however, the present study adds to the limited literature suggesting that stabilizing normally dynamic ovarian hormones, regardless of HC type, may be beneficial for cessation outcomes. It is also promising to note the differences in state anxiety scores between individuals using HCs versus those not using HCs. This provides preliminary evidence to warrant the further study of the utility of stabilizing hormone levels via HCs to reduce anxiety and potentially impact subsequent cessation attempts. Thus, it is important to further delineate the role of progestin-only and combined HCs in anxiety disorders and smoking cessation as potential treatment targets in future research.

There are limitations to the current study that are necessary to point out, including the cross-sectional design of the study, which precludes drawing conclusions on directionality of associations among variables. In addition, the study relied on a convenience sample with a restricted age range, relatively light smoking rate, and limited smoking experience (less than 4 years). These factors, including the relatively low FTCD scores, limit the external validity and generalizability of the findings to other populations of women who smoke. Importantly, the small effect sizes in the analyses caution that statistical significance may not indicate clinically meaningful group differences or associations among variables, especially given the large sample size. However, we would argue that identification of variables that account for even partial variance in cessation attempts is clinically meaningful and worthwhile, given the challenges women who smoke face when attempting to quit. There were a number of potentially important variables that we did not assess in the present study, including the current menstrual cycle phase of participants, other substance use, and detailed medication information. Further, the participants were self-selected to groups of HC use and type of HC versus random assignment. Thus, any significant differences might be a result of group variation at the outset rather than different because of the specific type of HC use. There may be other important differences between these groups that were not measured in the present study and might account for the differences observed in this sample. Additionally, participants were asked to identify their method of HC. This may have led to underreporting among participants as the more common name for HC, birth control, was not used. Finally, self-reported smoking status was not biochemically verified in the current study. However, it is unlikely participants would be motivated to misrepresent their smoking status, as participants were provided with course extra credit in return for their participation regardless of their smoking status. Also, existing evidence indicates that misreporting of smoking status is low among young adult samples such as participants in the current study (Ramo et al., 2011). Future research should further determine the role that HC use plays in smoking cessation, and whether it is beneficial to women attempting to quit smoking regarding nicotine withdrawal symptoms, and negative mood, as well as how it might affect longer-term abstinence.

## CRediT authorship contribution statement

**Shelby A. Stewart:** Project administration, Validation, Writing – original draft, Writing – review & editing. **MacKenzie R. Peltier:** Project administration, Validation, Writing – original draft, Writing – review & editing. **Melanie R. Roys:** Project administration, Validation, Writing – original draft, Writing – review & editing. **Amy L. Copeland:** Project administration, Validation, Writing – original draft, Writing – review & editing.

## Declaration of Competing Interest

The authors have no conflicts to declare.
